# Quality of life after patient-initiated vs physician-initiated response to symptom monitoring: the SYMPRO-Lung trial

**DOI:** 10.1093/jnci/djad159

**Published:** 2023-08-21

**Authors:** Nicole E Billingy, Vashti N M F Tromp, Neil K Aaronson, Rianne J A Hoek, Harm Jan Bogaard, Bregje D Onwuteaka-Philipsen, Lonneke van de Poll-Franse, Jacqueline G Hugtenburg, José Belderbos, Annemarie Becker-Commissaris, Corina J G van den Hurk, Iris Walraven, the SYMPRO-Lung Consortium, N C van Walree, K de Jaeger, S Samii, W Y Lam-Wong, F Koppe, J A Stigt, G J M Herder, A Welling, O C J Schuurbiers-Siebers, J M Smit, A J Staal-van den Brekel, W K de Jong

**Affiliations:** Department of Pulmonary Diseases, Cancer Center Amsterdam, Amsterdam Public Health Research Institute, Amsterdam University Medical Centers (UMC), Vrije Universiteit Amsterdam, Amsterdam, the Netherlands; Department of Clinical Pharmacology and Pharmacy, Amsterdam Public Health Research Institute, Amsterdam UMC, Vrije Universiteit Amsterdam, Amsterdam, the Netherlands; Division of Psychosocial Research and Epidemiology, The Netherlands Cancer Institute, Amsterdam, the Netherlands; Department of Pulmonary Diseases, Cancer Center Amsterdam, Amsterdam Public Health Research Institute, Amsterdam University Medical Centers (UMC), Vrije Universiteit Amsterdam, Amsterdam, the Netherlands; Department of Pulmonary Diseases, Cancer Center Amsterdam, Amsterdam Public Health Research Institute, Amsterdam University Medical Centers (UMC), Vrije Universiteit Amsterdam, Amsterdam, the Netherlands; Department of Public and Occupational Health, Amsterdam Public Health Research Institute, Cancer Center Amsterdam, Amsterdam UMC, Vrije Universiteit Amsterdam, the Netherlands; Division of Psychosocial Research and Epidemiology, The Netherlands Cancer Institute, Amsterdam, the Netherlands; Department of Research and Development, Netherlands Comprehensive Cancer Organisation (IKNL), Utrecht, the Netherlands; CoRPS—Center of Research on Psychological and Somatic Disorders, Department of Medical and Clinical Psychology, Tilburg University, Tilburg, the Netherlands; Department of Clinical Pharmacology and Pharmacy, Amsterdam Public Health Research Institute, Amsterdam UMC, Vrije Universiteit Amsterdam, Amsterdam, the Netherlands; Department of Radiation Oncology, Netherlands Cancer Institute, Amsterdam, the Netherlands; Department of Pulmonary Diseases, Cancer Center Amsterdam, Amsterdam Public Health Research Institute, Amsterdam University Medical Centers (UMC), Vrije Universiteit Amsterdam, Amsterdam, the Netherlands; Department of Research and Development, Netherlands Comprehensive Cancer Organisation (IKNL), Utrecht, the Netherlands; Department for Health Evidence, Radboud University Medical Center, Nijmegen, the Netherlands

## Abstract

**Background:**

Previous studies using patient-reported outcomes measures (PROMs) to monitor symptoms during and after (lung) cancer treatment used alerts that were sent to the health-care provider, although an approach in which patients receive alerts could be more clinically feasible. The primary aim of this study was to compare the effect of weekly PROM symptom monitoring via a reactive approach (patient receives alert) or active approach (health-care provider receives alert) with care as usual on health-related quality of life (HRQOL) at 15 weeks after start of treatment in lung cancer patients.

**Methods:**

The SYMPRO–Lung trial is a multicenter randomized controlled trial using a stepped wedge design. Stage I-IV lung cancer patients in the reactive and active groups reported PROM symptoms weekly, which were linked to a common alerting algorithm. HRQOL was measured by the EORTC QLQ-C30 at baseline and after 15 weeks. Linear regression analyses and effect size estimates were used to assess mean QOL–C30 change scores between groups, accounting for confounding.

**Results:**

A total of 515 patients were included (160 active group, 89 reactive group, 266 control group). No differences in HRQOL were observed between the reactive and active group (summary score: unstandardized beta [B] = 0.51, 95% confidence interval [CI] = -3.22 to 4.24, Cohen *d* effect size [ES] = 0.06; physical functioning: B = 0.25, 95% CI = -5.15 to 4.64, ES = 0.02). The combined intervention groups had statistically and clinically significantly better mean change scores on the summary score (B = 4.85, 95% CI = 1.96 to 7.73, ES = 0.57) and physical functioning (B = 7.00, 95% CI = 2.90 to 11.09, ES = 0.71) compared with the control group.

**Conclusions:**

Weekly PRO symptom monitoring statistically and clinically significantly improves HRQOL in lung cancer patients. The logistically less intensive, reactive approach may be a better fit for implementation.

Lung cancer is the second most common cancer diagnosed worldwide, and the most common cause of cancer deaths per year (1). Current treatment options include surgery, systemic treatment, and radiotherapy (2). Besides the disease itself, the treatment can cause a wide range of symptoms during and after treatment (3). These symptoms can negatively impact a patient’s health-related quality of life (HRQOL) and can also be a signal of disease recurrence or progression (4-6).

Previous randomized controlled trials (RCTs) investigated the effect of patient-reported outcomes (PRO) symptom monitoring on overall survival and HROQL. These studies reported significantly better HRQOL and overall survival compared with care as usual (7-13). Nonetheless, these significant results were obtained within the controlled conditions of RCTs, using an ‘active’ approach in which dedicated research nurses were available 24 hours per day, 7 days per week to monitor the symptoms. Additionally, extra nursing staff was funded to educate, train, and support participants in using the patient-reported outcome measure (PROM) (7,9,11). Such a logistically intensive intervention may not be feasible within real-world clinical practice. Basch et al. (11) noted that, despite their positive results, widespread adoption of the intervention has not been forthcoming in part because of the substantial efforts required from the staff.

A more ‘reactive’ patient-centered approach in which the patient receives the alert and is encouraged to contact the hospital following an alert might be equally effective while significantly lowering the workload for the health-care providers. The primary aim of the SYMPRO–Lung study was to compare the effect of weekly online PRO symptom monitoring via a reactive approach (patient receives alert) or an active approach (health-care provider receives alert) with care as usual on the HRQOL of lung cancer patients at 15 weeks after start of treatment.

## Methods

### Study design and study sample

We conducted a multicenter RCT using a stepped wedge design, the details of which have been reported previously (14). Briefly, (non)–small cell lung cancer patients with stage I-IV, who were starting a treatment with radiotherapy, surgery, chemotherapy, immunotherapy, and/or targeted therapy were eligible to participate.

The stepped wedge cluster randomized design involved sequential transition of the participating hospitals in a randomized order. Over a period of 16 months, 13 Dutch hospitals (3 academic and 10 nonacademic) consecutively switched from care as usual (the control group) to an intervention period (see Supplementary Figure 1, available online). The inclusion period ran from October 2019 until September 2021. Because of the COVID-19 pandemic, 1 hospital was replaced by a new hospital and recruitment was delayed and thereby extended by a few weeks to several months, depending on the resources available per hospital. Data collection ended in January 2022.

All patients provided written informed consent upon inclusion. The study was approved by the institutional review board and medical ethical committee of the Amsterdam University Medical Centers (UMC), location Vrije Univeristeit medical center (VUmc), as well as by the review boards of all participating centers. The study was registered in the Netherlands trial register NL7897.

### Intervention

There were 2 intervention groups differing in the way the alerts were handled; 1 reactive group in which patients themselves received an alert via a pop-up notification and (secure) e-mail containing the advice to contact the hospital within 24 hours on weekdays; 1 active group in which the health-care providers received an alert via a (secure) e-mail instructing them to contact the patient within 24 hours on weekdays (during office hours). Directly following initiation of an alert, patients were asked if the symptoms were still present and, if not, that they would opt-out of being contacted by their health-care provider. This was done to prevent unnecessary phone calls from their health-care provider, because the Patient-Reported Outcomes version of the Common Toxicity Criteria for Adverse Events (PRO-CTCAE) (15,16) questions referred to the past 7 days. If the symptom was still present, the health-care provider contacted the patient to perform triage, give tailored advice, and intervene when needed. The health-care provider who contacted the patient was often a specialized nurse. After all alerts, the health-care providers were asked to complete a single question to record what clinical actions were taken in direct response to the alert.

Both intervention groups were given access to the online application on weekdays to report their symptoms weekly. Patients were able to contact the study staff via telephone or e-mail if they experienced problems using the application.

**Figure 1.**
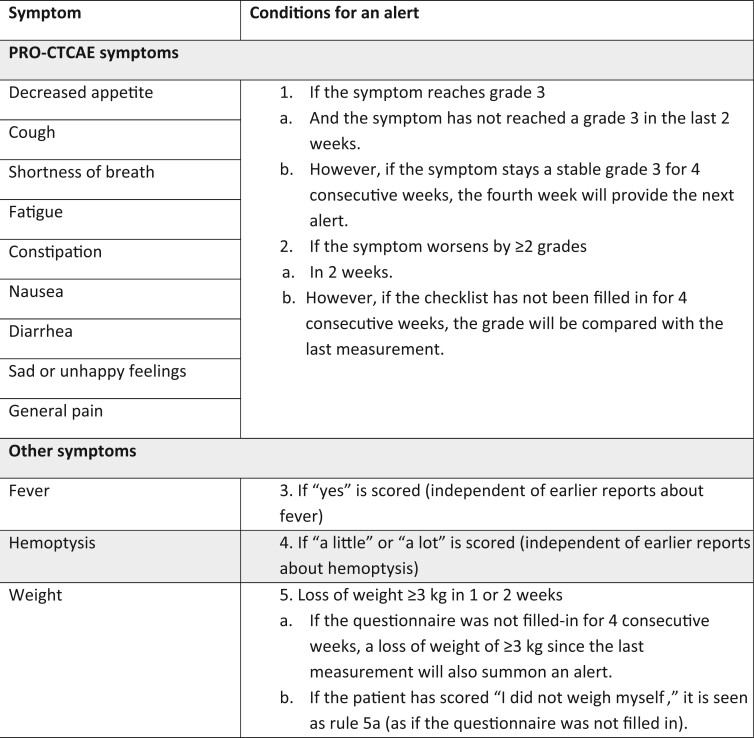
Set of predefined conditions that prompted the web application to create an alert. PRO-CTCAE = Patient-Reported Outcomes–Common Terminology Criteria for Adverse Events.

The lung cancer subset (17) from the PRO-CTCAE (15,16,18) was used to monitor symptoms. The symptoms can be found in Figure 1, including information on the predefined conditions that had to be met that triggered an alert indicating the need for intervention.

### Control group

Patients in the control group received care as usual. This consisted of the standard procedure at their hospital for assessing and documenting symptoms (predominantly discussing symptoms with their health-care provider during outpatient visits and a documentation in their medical record).

## Study measures

### Primary outcomes

The primary outcome was the mean change from baseline to 15 weeks (T1) after the start of treatment in HRQOL as assessed by the European Organisation for Research and Treatment of Cancer Quality of Life Questionnaire (EORTC QLQ-C30) (19,20). The 15 weeks’ time point was considered as our first primary endpoint where the effects were measurable and most patients survived. The EORTC QLQ-C30 scores were calculated according to the guidelines (21). Our main HRQOL endpoints were physical functioning and the summary score. We chose both scales because they have recently shown to have more prognostic value than the global health status scale (22).

Additional information on the EORTC QLQ-C30 scales, interpretation of clinically relevant change scores, our secondary outcomes, and sensitivity analyses can be found in the Supplementary Methods section of the Supplementary Materials (available online).

### Statistical analysis

The trial protocol included a sample size of 584, yielding 80% power to detect a Cohen’s *d* effect size (ES) of less than 0.2 with a 1-sided alpha set at 0.05, between the reactive and active intervention group (14). To demonstrate noninferiority between the 2 groups, 146 patients per intervention group were needed.

Data were analyzed on an intention-to-treat basis. Patient characteristics are presented as proportions: mean (SD). Differences in baseline characteristics and HRQOL were compared using independent samples *t* tests (continuous variables, if normally distributed) and χ^2^ tests (categorical variables). The proportion of patients in each group who experienced (clinically relevant) changes in HRQOL scores were compared using Pearson χ^2^ tests.

Comparisons were made between the reactive and active intervention groups and between the combined intervention groups vs the control group. Between-groups differences in mean changes in QLQ-C30 scores from baseline to 15 weeks after start of treatment were analyzed using multivariable linear regression analyses. Because we only compared 2 time points, multivariable linear regression analyses were conducted, adjusted for all confounding variables at baseline: baseline QLQ-C30 scale score, transfer sequence of the hospitals, and the baseline characteristics that were significantly different between the intervention groups. Group differences in mean change scores over time between the groups were accompanied by Cohen’s *d* effect size (23). The trial protocol stated that an effect size of less than 0.2 would demonstrate noninferiority between the active and reactive intervention group. An effect size of 0.4 was considered as the minimum HRQOL benefit to demonstrate superiority of the PRO symptom monitoring compared with the control group (14).

All analyses were performed in SPSS version 28.0. All tests were 2-sided with an assumed statistical significance level of a *P *value less than* *.05.

## Results

**Figure 2.**
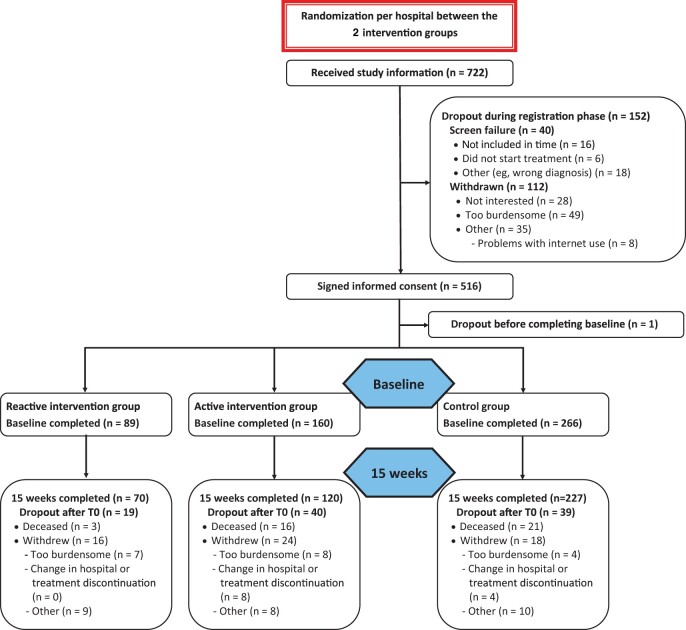
CONSORT diagram of the Symptom Reporting with Patient-Reported Outcomes–Lung trial.

A total of 515 patients completed the baseline questionnaire (Figure 2): 266 in the control group, 160 in the active intervention group, and 89 in the reactive intervention group. Completion rates of the HRQOL questionnaire 15 weeks after start of treatment were 81% (n = 417), with statistically significant more patients in the control group completing their questionnaire compared with the active and reactive intervention groups (control n = 227, 85%; active n = 120, 75%; reactive n = 70, 79%; *P *=* *.026).

### Baseline characteristics

Most baseline sociodemographic and clinical characteristics were balanced across groups (Table 1). The most pronounced exception between the intervention group and the control group was the difference in stage distribution: the intervention group had statistically significantly more stage IV patients compared with the control group (56% vs 44%; *P *=* *.004). Other exceptions were in histology and treatment (Table 1).

**Table 1.** Baseline characteristics

Patient characteristics	Total study sample	Control group	Intervention group		Active intervention	Reactive intervention	
	No. (%)	No. (%)	No. (%)	*P*	No. (%)	No. (%)	*P*
Total	515	266	249		160	89	
Age, mean (SD), y	65.4 (9.4)	66.0 (9.2)	64.8 (9.7)	.162	64.6 (10.5)	65.3 (8.0)	.555
Sex, % male	266 (51.7)	139 (52.3)	127 (51.0)	.776	84 (52.5)	43 (48.3)	.527
Education				.083			.066
Primary education	230 (44.7)	121 (45.5)	109 (43.8)		77 (48.1)	32 (36.0)	
High school and vocational education	145 (28.2)	83 (31.2)	62 (24.9)		40 (25.0)	22 (24.7)	
College, university	136 (26.4)	60 (22.6)	76 (30.5)		41 (25.6)	35 (39.3)	
Missing	4 (0.8)	2 (0.8)	2 (0.8)		2 (1.3)	0 (0)	
Living status				.913			.523
Alone	113 (21.9)	59 (22.2)	54 (21.7)		33 (20.6)	21 (23.6)	
Together, with another adult	399 (77.5)	206 (77.4)	193 (77.5)		127 (79.4)	66 (74.2)	
Missing	3 (0.6)	1 (0.4)	2 (0.8)		0 (0)	2 (2.2)	
Work, % employed	144 (28.0)	66 (24.8)	78 (31.3)	.100	50 (31.3)	28 (31.5)	.973
ECOG Performance Status				.396			.036
0	210 (40.8)	104 (39.1)	106 (42.6)		60 (37.5)	46 (51.7)	
1	263 (51.1)	143 (53.8)	120 (48.2)		81 (50.6)	39 (43.8)	
2	42 (8.2)	19 (7.1)	23 (9.2)		19 (11.9)	4 (4.5)	
Other comorbidities, % Yes	231 (44.9)	122 (45.9)	109 (43.8)	.634	76 (47.5)	33 (37.1)	.112
Histology				<.001			.017
Nonadenocarcinoma, nonsquamous	46 (8.9)	30 (11.3)	16 (6.4)		12 (7.5)	4 (4.5)	
Adenocarcinoma, M−	257 (49.9)	120 (45.1)	137 (55.0)		84 (52.5)	53 (59.6)	
Adenocarcinoma, M+	40 (7.8)	14 (5.3)	26 (10.4)		24 (15.0)	2 (2.2)	
Squamous cell carcinoma	85 (16.5)	51 (19.2)	34 (13.7)		19 (11.9)	15 (16.9)	
Small cell lung cancer	45 (8.7)	19 (7.1)	26 (10.4)		17 (10.6)	9 (10.1)	
No histology confirmed	42 (8.2)	32 (12.0)	10 (4.0)		4 (2.5)	6 (6.7)	
Stage distribution				.004			.035
I	75 (14.6)	52 (19.5)	23 (9.2)		13 (8.1)	10 (11.1)	
II	33 (6.4)	16 (6.0)	17 (6.8)		8 (5.0)	9 (10.1)	
III	150 (29.1)	80 (30.1)	70 (28.1)		39 (24.4)	31 (34.8)	
IV	257 (49.9)	118 (44.4)	139 (55.8)		100 (62.5)	39 (43.8)	
Previous treatment for lung cancer, % yes^a^	192 (37.3)	90 (33.8)	102 (41.0)	.095	61 (38.1)	41 (46.1)	.222
Treatment plan				<.001			.015
Immunotherapy	74 (14.4)	45 (16.9)	29 (11.6)		22 (13.8)	7 (7.9)	
Immunotherapy combined	87 (16.9)	44 (16.5)	43 (17.3)		29 (18.1)	14 (15.7)	
Radiotherapy (combined)	90 (17.5)	62 (23.3)	28 (11.2)		12 (7.5)	16 (18.0)	
Chemotherapy (combined)	197 (38.3)	92 (34.6)	105 (42.2)		64 (40.0)	41 (46.1)	
Targeted therapy	37 (7.2)	9 (3.4)	28 (11.2)		24 (15.0)	4 (4.5)	
Surgery	30 (5.8)	14 (5.3)	16 (6.4)		9 (5.6)	7 (7.9)	

a Immunotherapy combined: immunotherapy (combined with chemotherapy or chemoradiation and/or surgery and/or targeted therapy. Radiotherapy combined: radiotherapy (combined with surgery). Chemotherapy combined: chemotherapy or chemoradiation (combined with radiotherapy, and/or targeted therapy and/or surgery). ECOG = Eastern Cooperative Oncology Group; M− = no targetable driver mutation or unknown status; M+ = targetable driver mutation.

Between the reactive and active intervention groups, exceptions were found in the same baseline characteristics (stage distribution, histology, and treatment). In addition, the reactive group had statistically significantly fewer patients with an Eastern Cooperative Oncology Group (ECOG) performance status score of 0 compared with the active intervention group (52% vs 38%; *P *=* *.036). See Table 1 for a detailed overview of the baseline characteristics.

Patients who did not complete the follow-up questionnaire had a statistically significantly worse ECOG performance status and stage distribution (data not shown). However, no differences were observed between patients in the control vs the intervention group.

### Symptom reporting and alert interventions

Of the 249 intervention patients, 244 (98%) completed at least 1 weekly symptom checklist. In total, 2412 symptom checklists were completed by the intervention group (see Supplementary Table 1 for completion rates per week, available online), with a median of 11 (4.34) checklists per patient. This resulted in 684 (28%) alerts, with a median of 2 (2.00) per patient. Completion rates (active 66% vs reactive 70%; *P *=* *.138) and alert rates (active 28% vs reactive 29%; *P *=* *.082) did not differ between the 2 intervention groups. The most common symptoms that triggered an alert were fatigue (33%), pain (28%), and constipation (21%). For 237 (35%) alerts, patients did not request any follow-up action. No statistically significant differences were observed in the proportion of alerts between the active (49%) and reactive intervention (50%) groups, for which direct action was necessary. Thus, for 447 alerts (active 292 [68%], reactive 155 [61%]), direct action was taken in response to the alert. These actions were mostly telephone contact (74%) in which advice was given or supportive medication was prescribed or altered. Use of the application was recommended by 94% of the patients.

## Primary HRQOL outcomes

### Noninferiority analyses (reactive vs active intervention group)

The reactive and active intervention groups were comparable in terms of baseline summary score (mean = 76.6 [SD = 16.0] in the reactive group; mean = 75.8 [SD = 14.1] in the active group; *P *=* *.728; see Table 2). For physical functioning, the reactive group had statistically significantly better mean physical functioning at baseline compared with the active group (mean = 79.1 [SD = 18.3] vs mean = 72.4 [SD = 21.6]; *P *=* *.032).

**Table 2.** Multivariable linear regression analysis for health-related quality of life from baseline to 15 weeks reactive vs active intervention group including confounders

EORTC QLQ-C30^a^ n = 190 (70 reactive, 120 active)					Multivariable analyses^c^			
		Mean baseline	Mean 15 weeks	Mean change score and clinical relevance^b^	B (SE)	95% CI	*P*	ES
Functioning scales								
QLQ summary score	Reactive intervention group	76.58	80.38	+3.80	0.51 (1.89)	−3.22 to 4.24	.788	0.06
				n/a				
	Active intervention group	75.80	80.29	+4.49				
				n/a				
Physical functioning	Reactive intervention group	79.05	76.57	−2.48	−0.25 (2.48)	−5.15 to 4.64	.919	0.02
				Trivial				
	Active intervention group	72.39	74.50	+2.11				
				Small improvement				
Global health status	Reactive intervention group	66.43	68.93	+2.50	1.75 (2.25)	−2.70 to 6.20	.437	0.14
				Trivial				
	Active intervention group	62.08	66.81	+4.73				
				Trivial				
Role functioning	Reactive intervention group	66.67	65.95	−0.72	0.16 (3.73)	−7.21 to 7.52	.967	0.01
				Trivial				
	Active intervention group	63.61	66.81	+3.20				
				Trivial				
Emotional functioning	Reactive intervention group	76.90	78.71	+1.81	−4.13 (2.69)	−9.43 to 1.18	.126	0.37
				Trivial				
	Active intervention group	74.24	81.67	+7.43				
				Small improvement				
Cognitive functioning	Reactive intervention group	82.14	83.10	+0.96	−0.90 (2.43)	−5.69 to 3.90	.713	0.08
				Trivial				
	Active intervention group	84.58	85.97	+1.39				
				Trivial				
Social functioning	Reactive intervention group	70.71	77.62	+6.91	−0.77 (3.17)	−7.04 to 5.49	.808	0.04
				Small improvement				
	Active intervention group	73.61	80.56	+6.95				
				Small improvement				
Symptom scales								
Fatigue	Reactive intervention group	34.67	33.33	+1.34	−0.47 (3.24)	−6.87 to 5.93	.884	0.03
				Trivial				
	Active intervention group	38.33	34.63	+3.70				
				Trivial				
Nausea and vomiting	Reactive intervention group	10.24	7.14	+3.10	−0.30 (2.12)	−4.50 to 3.89	.887	0.02
				Small improvement				
	Active intervention group	7.92	6.39	+1.53				
				Trivial				
Pain	Reactive intervention group	26.90	15.71	+11.19	−4.54 (3.58)	−11.60 to 2.52	.206	0.24
				Medium improvement				
	Active intervention group	26.25	19.44	+6.81				
				Small improvement				
Dyspnea	Reactive intervention group	30.95	30.48	+0.47	−3.97 (3.85)	−11.58 to 3.63	.304	0.23
				Trivial				
	Active intervention group	34.17	33.89	+0.28				
				Trivial				
Insomnia	Reactive intervention group	26.67	20.95	+5.72	4.40 (3.79)	−3.09 to 11.89	.248	0.21
				Small improvement				
	Active intervention group	27.22	16.67	+10.55				
				Medium improvement				
Appetite loss	Reactive intervention group	21.90	16.19	+5.71	−3.42 (3.81)	−10.94 to 4.10	.370	0.19
				Trivial				
	Active intervention group	16.94	15.83	+1.11				
				Trivial				
Constipation	Reactive intervention group	17.62	9.52	+8.1	4.08 (3.13)	−2.11 to 10.28	.195	0.16
				Small improvement				
	Active intervention group	23.06	8.61	+14.45				
				Medium improvement				
Diarrhea	Reactive intervention group	10.95	8.10	+2.85	0.28 (2.78)	−5.20 to 5.75	.921	0.02
				Trivial				
	Active intervention group	9.17	8.61	+0.56				
				Trivial				
Financial difficulties	Reactive intervention group	9.52	8.10	+1.42	−2.91 (2.62)	−8.07 to 2.26	.268	0.34
				Trivial				
	Active intervention group	5.83	8.61	−2.78				
				Small deteriorations				

a EORTC quality of life score and functioning scales: higher score = better quality of life and/or functioning. EORTC symptom scales: higher score = worse symptoms. T1 = 15 weeks; B = unstandardized beta; ES = Cohen’s *d* effect size: 0.2 = small; 0.5 = medium; 0.8 = large clinical relevance. *P* values for between-group comparisons. CI = confidence interval; EORTC QLQ-C30 = European Organisation for Research and Treatment of Cancer Quality of Life Questionnaire.

b Clinical relevance according to guidelines of Cocks et al. (33) for longitudinal within-group differences. To date, no clinical relevant thresholds have been established for the Summary score.

c Multivariable analyses were controlled for histology, treatment, cancer stage, Eastern Cooperative Oncology Group Performance Status, baseline score, and transfer sequence of the hospitals.

After controlling for confounders, between the reactive and active intervention group in the multivariable linear regression analyses, no statistically or clinically significant differences were found between the summary score (unstandardized beta [B] = 0.51, 95% confidence interval [CI] = -3.22 to 4.24, ES = 0.06; *P *=* *.788) or physical functioning scale (B = -0.25, 95% CI = -5.15 to 4.64, ES = 0.02; *P *=* *.919) (see Table 2). Effect sizes were less than 0.2. Therefore, the criteria for noninferiority between the reactive and active intervention groups were met.

### Superiority analyses (intervention vs control group)

The intervention and control groups were comparable in terms of baseline summary score and physical functioning (Table 3). Mean baseline summary score was 76.1 (SD = 14.8) in the intervention group and 76.2 (SD = 14.1) in the control group (*P *=* *.918). Mean baseline physical functioning score was 74.8 (SD = 20.7) and 73.5 (SD = 19.5; *P *=* *.490), respectively.

**Table 3.** Multivariable linear regression analysis for health-related quality of life from baseline to 15 weeks intervention vs control group including confounders

EORTC QLQ-C30^a^ n = 417 (intervention 190, control 227)					Multivariable analyses^c^			
		Mean baseline	Mean 15 weeks	Mean change score and clinical relevance^b^	B (SE)	95% CI	*P*	ES
Functioning scales								
QLQ summary score	Intervention group	76.08	80.32	+4.24	4.85 (1.47)	1.96 to 7.73	.001	0.57
				n/a				
	Control group	76.23	75.85	−0.38				
				n/a				
Physical functioning	Intervention group	74.84	75.26	+0.42	7.00 (2.08)	2.90 to 11.09	<.001	0.71
				Trivial				
	Control group	73.48	67.75	−5.73				
				Small deterioration				
Global health status^d^	Intervention group	63.68	67.59	+3.91	3.40 (2.02)	−0.56 to 7.36	.092	0.25
				Trivial				
	Control group	62.02	63.13	+1.11				
				Trivial				
Role functioning	Intervention group	64.74	66.49	+1.75	9.44 (3.03)	3.48 to 15.40	.002	0.49
				Trivial				
	Control group	62.56	56.61	−5.95				
				Trivial				
Emotional functioning^d^	Intervention group	75.22	80.61	+5.39	2.96 (1.99)	−0.95 to 6.87	.138	0.31
				Trivial				
	Control group	72.90	75.81	+2.91				
				Trivial				
Cognitive functioning	Intervention group	83.68	84.91	+1.23	3.39 (2.07)	−0.68 to 7.45	.102	0.36
				Trivial				
	Control group	84.14	81.57	−2.57				
				Small deterioration				
Social functioning^d^	Intervention group	72.54	79.47	+6.93	10.65 (2.65)	5.44 to 15.86	<.001	0.65
				Small improvement				
	Control group	74.78	71.68	−3.10				
				Trivial				
Symptom scales								
Fatigue	Intervention group	37.02	34.15	+2.87	−8.59 (2.68)	−13.85 to -3.32	.001	0.52
				Trivial				
	Control group	38.86	41.75	−2.89				
				Trivial				
Nausea and vomiting	Intervention group	8.77	6.67	+2.10	−2.85 (1.81)	−6.43 to 0.72	.118	0.22
				Trivial				
	Control group	8.59	8.15	+0.44				
				Trivial				
Pain	Intervention group	26.49	18.07	+8.42	−4.28 (2.72)	−9.63 to 1.07	.116	0.24
				Small improvement				
	Control group	22.61	20.78	+1.83				
				Trivial				
Dyspnea	Intervention group	32.98	32.63	+0.35	−10.13 (3.22)	−16.47 to -3.79	.002	0.58
				Trivial				
	Control group	34.65	42.73	−8.17				
				Small deterioration				
Insomnia	Intervention group	27.02	18.25	+8.77	2.48 (2.83)	−3.08 to 8.04	.381	0.12
				Small improvement				
	Control group	30.54	20.41	+10.13				
				Medium improvement				
Appetite loss	Intervention group	18.77	15.96	+2.81	−0.07 (2.79)	−5.55 to 5.41	.981	<0.01
				Trivial				
	Control group	19.68	17.18	+2.50				
				Trivial				
Constipation	Intervention group	21.05	8.95	+12.1	−10.81 (2.55)	−15.82 to -5.79	<.001	0.48
				Medium improvement				
	Control group	15.42	14.10	+1.32				
				Trivial				
Diarrhea	Intervention group	9.82	8.42	+1.4	1.42 (1.92)	−2.37 to 5.20	.462	0.10
				Trivial				
	Control group	6.31	6.46	−0.15				
				Trivial				
Financial difficulties^d^	Intervention group	7.19	8.42	−1.23	−0.56 (1.70)	−3.91 to 2.78	.740	0.08
				Trivial				
	Control group	5.16	6.05	−0.89				
				Trivial				

a EORTC quality-of-life score and functioning scales: higher score = better quality of life and/or functioning. EORTC symptom scales: higher score = worse symptoms. T1 = 15 weeks; B = unstandardized beta; SE = Cohen’s *d* effect size = 0.2 = small; 0.5 = medium; 0.8 = large clinical relevance. *P* values for between-group comparisons. CI = confidence interval; EORTC QLQ-C30 = European Organisation for Research and Treatment of Cancer Quality of Life Questionnaire.

b Clinical relevance according to guidelines of Cocks et al. (33) for longitudinal within-group differences. To date, no clinical relevant thresholds have been established for the Summary score.

c Multivariable analyses were controlled for histology, treatment, cancer stage, baseline score, and transfer sequence of the hospitals.

d n = 416.

**Figure 3.**
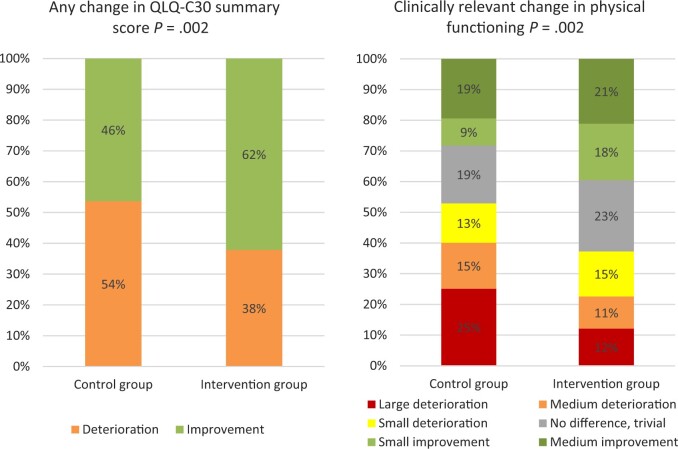
Proportion of patients with health-related quality of life changes at 15 weeks (T1) compared with baseline. For the clinically relevant change in physical functioning Cock et al. (33) guidelines for within-group changes were used: improvements: medium >7, small 7-2; no difference or trivial 2 to -5; deteriorations: small -5 to -10, medium -10 to -17, large <-17. *P* values were calculated using Pearson χ^2^ tests comparing the control group vs the intervention group.

The summary score improved (to any degree) from baseline to 15 weeks after start of treatment among statistically significantly more patients in the intervention group than in the control group (62% vs 46%) and deteriorated among fewer patients (38% vs 54%; *P *=* *.002; Figure 3). Physical functioning showed more clinically relevant improvement in more patients in the intervention group (40% vs 28%) and deteriorated in fewer patients (37% vs 53%; *P *=* *.002) than in the control group (Figure 3).

After controlling for confounders, both the summary score (B = 4.85, 95% CI = 1.96 to 7.73, ES = 0.57; *P *=* *.001) and physical functioning (B = 7.00, 95% CI = 2.90 to 11.09, Cohen *d* = 0.71; *P *<* *.001) improved statistically and clinically significantly more in the intervention group compared with the control group (Table 3). Therefore, the criteria for superiority of the intervention over the control group were reached.

### Secondary HRQOL

For the majority of the remaining QLQ-C30 scale scores, mean differences between the reactive and active intervention groups also reached an effect size below our threshold of 0.2, indicating noninferiority (Table 2).

The mean (change) scores for all functioning and symptom scales can be found in Table 3 for the superiority analyses. The multivariable linear regression analyses (adjusted for confounders) also showed statistically significantly more improvement on multiple scales (role and social functioning, fatigue, dyspnea, and constipation) for the intervention group compared with the control group, with effect sizes above our set threshold of 0.4 for defining superiority of the intervention group over the control group (Table 3). Effect sizes were almost all medium for the above-mentioned scales and thus clinically relevant. All these scales had clinically relevant within-group differences in the intervention group that were similar to, or better than, the control group (Table 3). For the above-mentioned scales that showed statistical significance, the proportion of patients experiencing clinically relevant improvement or deterioration can be found in the Supplementary Figure 2 (available online).

### Sensitivity analyses (multiple imputation)

Multiple imputation analyses did not result in statistically significantly different associations (see Supplementary Table 2, available online). Between-group differences in mean change scores were a little smaller, but still statistically significant and clinically relevant.

## Discussion

In this trial assessing the effect of patient-reported symptom monitoring on HRQOL in lung cancer patients, we observed that a reactive approach (patient receives alert) and an active approach (health-care provider receives alert) are equally effective on HRQOL. Furthermore, we confirmed that patient-reported outcomes symptom monitoring was significantly associated with improved HRQOL across almost all scales compared with care as usual. Because of the increasing time demands placed on health-care providers (24,25), the reactive, more patient-centered approach can facilitate sustainable implementation of symptom monitoring within daily clinical practice.

Our findings are consistent with earlier studies on PRO symptom monitoring, which reported similar results with respect to HRQOL in metastatic cancer patients (7,11,26), curatively treated patients (27), and lung cancer patients (9). The recent RCT conducted by Basch et al. (11) in metastatic oncology patients with a logistically intense active approach (eg, reminder calls if patients did not complete their weekly symptom checklist within 72 hours) resulted in a difference in mean change of 2.4 for the QLQ summary score and 2.5 points for physical functioning. Compared with this latter study, our results showed even larger effects (4.9 and 7.0 points, respectively) with the reactive intervention group. These differences were still present when we restricted our analyses to the subset of patients with metastatic disease (results not shown). It is also noteworthy that improvements in dyspnea, fatigue, and constipation were observed. These 3 symptoms were among the 5 symptoms that most often triggered a symptom alert and are symptoms that patients indicate they would like to reduce (28-30). Our findings emphasize the effectiveness of using PROMs for monitoring symptoms in patients in all cancer stages, receiving all treatment options. It is important for lung cancer patients, a patient group where there is still so much progress to be made. Several previous studies have investigated the use of PROs linked to an alert system for lung cancer patients (9,31); these studies were focused primarily on overall survival and hospitals visits and/or care consumption. In these studies, positive results were reported in terms of overall survival, fewer emergency room visits and admissions, and shorter hospital stays. However, these studies did not report (extensively) on HRQOL outcomes. Taken together, our results and those of earlier studies yield sufficient evidence to recommend routine implementation of PRO–based symptom monitoring to improve a range of clinical and HRQOL outcomes.

Several limitations of our study should be noted. First, random assignment at the level of hospital resulted in some differences between the study groups with regard to the characteristics of the patients recruited. However, we were able to adjust for these baseline differences and still observed large effect sizes. Second, primarily because of the COVID-19 pandemic, we did not reach our intended sample size in the reactive intervention group. However, when taking the observed differences into account, using a noninferiority margin of 0.2*standard deviation, a power of 59% was reached for the summary score and 99% power for physical functioning. We believe that this provides sufficient evidence that the 2 approaches yield comparable HRQOL outcomes. Third, 58 (11%) still living patients did not complete their T1 HRQOL questionnaire of which 40 (69%) were from the intervention group. The dropout rate could be a sign of selective dropout in the intervention group due to worse health statuses. Therefore, it might be that the reported effects are a slight overrepresentation. However, the patients for whom data were missing in the intervention and control groups were similar in their baseline health status characteristics. Thus, we assume that this effect is marginal. Additionally, only 15 (25%) patients in the intervention groups reported that using the app was too burdensome compared with 4 (10%) patients in the control group (Figure 2). Nonetheless, the higher dropout rate in the intervention group was not entirely because of questionnaire burden. Other reasons also mentioned were that patients no longer saw the point in participating because their cancer had worsened or improved (6%).

There may have been selection bias during the recruitment phase because the application was only available on an electronic device. However, of the 112 patients who received study information and declined participation, only 8 (7%) patients reported that it was because of the electronic aspect of the intervention. Although, the minimal role of internet access as a barrier is reinforced by the fact that in 2022, 96% of the Dutch population aged 65-75 years and 80% of age those aged 75 years and older made use of the internet (32). The mean age of our sample was 65 years, and 16% of the patients were aged 75 years or older.

A major strength of our study is that we conducted a head-to-head comparison of a reactive and an active approach to web-based symptom monitoring. By including hospitals in our study from throughout the Netherlands and recruiting patients with all stages of lung cancer who were receiving all types of treatment, our study population is comparable to the real-world population, improving generalizability. Finally, the multicenter, stepped wedge cluster design made it possible to implement the intervention in daily clinical practice, specifically adapting the logistics to the work method of each participating hospital. This increases the relevance of our findings to the real world of clinical care for patients with lung cancer. Future research will further reveal the effect of PRO symptom monitoring on long-term HRQOL and our secondary outcomes overall survival, cost-effectiveness, usefulness, and implementation fidelity of the application.

In conclusion, our results indicate that PRO symptom monitoring statistically and clinically significantly improves HRQOL in lung cancer patients. The reactive approach was equally effective to the standard active approach. Therefore, the reactive approach, which is logistically less intensive and less demanding for health-care providers, may be a better fit for efficient implementation within real-world clinical practice. Health-care providers can choose the symptom-altering approach that best suits their situation and that of their patients.

## Supplementary Material

djad159_Supplementary_Data

## Data Availability

Data from the SYMPRO–Lung study are available upon request and permission by the principal investigators (PIs). Please contact Nicole Billingy (n.billingy@amsterdamumc.nl) to submit your proposal. She will coordinate the request to the PIs. Use of the data by third parties will then be reviewed for each research question.

## References

[1] Sung H , Ferlay J, Siegel RL, et al Global cancer statistics 2020: GLOBOCAN estimates of incidence and mortality worldwide for 36 cancers in 185 countries. CA Cancer J Clin. 2021;71(3):209-249. 33538338 10.3322/caac.21660

[2] National Cancer Institute *Non-Small Cell Lung Cancer Treatment (PDQ^®^)–Health Professional Version.* https://www.cancer.gov/types/lung/hp/non-small-cell-lung-treatment-pdq#_485751_toc. Accessed March 17, 2022.

[3] Cooley ME. Symptoms in adults with lung cancer. A systematic research review. J Pain Symptom Manage. 2000;19(2):137-153. 10699541 10.1016/s0885-3924(99)00150-5

[4] Basch E , Jia X, Heller G, et al Adverse symptom event reporting by patients vs clinicians: relationships with clinical outcomes. J Natl Cancer Inst. 2009;101(23):1624-1632. 19920223 10.1093/jnci/djp386 PMC2786917

[5] Burkhart PV , Sabate E. Adherence to long-term therapies: evidence for action. J Nurs Scholarsh. 2003;35(3):207. 14562485

[6] Denis F , Viger L, Charron A, et al Detection of lung cancer relapse using self-reported symptoms transmitted via an internet web-application: pilot study of the sentinel follow-up. Support Care Cancer. 2014;22(6):1467-1473. 24414998 10.1007/s00520-013-2111-1

[7] Basch E , Deal AM, Kris MG, et al Symptom monitoring with patient-reported outcomes during routine cancer treatment: a randomized controlled trial. J Clin Oncol. 2016;34(6):557-565. 26644527 10.1200/JCO.2015.63.0830 PMC4872028

[8] Basch E , Deal AM, Dueck AC, et al Overall survival results of a trial assessing patient-reported outcomes for symptom monitoring during routine cancer treatment. JAMA. 2017;318(2):197-198. 28586821 10.1001/jama.2017.7156 PMC5817466

[9] Denis F , Lethrosne C, Pourel N, et al Randomized trial comparing a web-mediated follow-up with routine surveillance in lung cancer patients. J Natl Cancer Inst. 2017;109(9):djx029. doi:10.1093/jnci/djx029. 28423407

[10] Denis F , Basch E, Septans AL, et al Two-year survival comparing web-based symptom monitoring vs routine surveillance following treatment for lung cancer. JAMA. 2019;321(3):306-307. 30667494 10.1001/jama.2018.18085 PMC6439676

[11] Basch E , Schrag D, Henson S, et al Effect of electronic symptom monitoring on patient-reported outcomes among patients with metastatic cancer: a randomized clinical trial. JAMA. 2022;327(24):2413-2422. doi:10.1001/jama.2022.9265. 35661856 PMC9168923

[12] Mooney KH , Beck SL, Wong B, et al Automated home monitoring and management of patient‐reported symptoms during chemotherapy: results of the symptom care at home RCT. Cancer Medicine. 2017;6(3):537-546. 28135050 10.1002/cam4.1002 PMC5345623

[13] Nipp RD , Horick NK, Deal AM, et al Differential effects of an electronic symptom monitoring intervention based on the age of patients with advanced cancer. Ann Oncol. 2020;31(1):123-130. 31912785 10.1016/j.annonc.2019.09.003 PMC7497788

[14] Billingy NE , Tromp VN, Veldhuijzen E, et al SYMptom monitoring with Patient-Reported Outcomes using a web application among patients with Lung cancer in the Netherlands (SYMPRO-Lung): study protocol for a stepped-wedge randomised controlled trial. BMJ Open. 2021;11(9):e052494. 10.1136/bmjopen-2021-052494 PMC8438957 34518276

[15] Basch E , Reeve BB, Mitchell SA, et al Development of the National Cancer Institute’s patient-reported outcomes version of the common terminology criteria for adverse events (PRO-CTCAE). J Natl Cancer Inst. 2014;106(9):dju244. doi:10.1093/jnci/dju244. PMC4200059 25265940

[16] Dueck AC , Mendoza TR, Mitchell SA, et al; for the National Cancer Institute PRO-CTCAE Study Group. Validity and reliability of the US National Cancer Institute’s Patient-Reported Outcomes Version of the Common Terminology Criteria for Adverse Events (PRO-CTCAE). JAMA Oncol. 2015;1(8):1051-1059. 26270597 10.1001/jamaoncol.2015.2639 PMC4857599

[17] Veldhuijzen E , Walraven I, Belderbos J. Selecting a subset based on the Patient-Reported Outcomes Version of the Common Terminology Criteria for Adverse Events for patient-reported symptom monitoring in lung cancer treatment: mixed methods study. JMIR Cancer. 2021;7(3):e26574. 34519658 10.2196/26574 PMC8479599

[18] Veldhuijzen E , Walraven I, Mitchell SA, et al Dutch translation and linguistic validation of the US National Cancer Institute’s Patient-Reported Outcomes Version of the Common Terminology Criteria for Adverse Events (PRO-CTCAE™). J Patient-Rep Outcomes. 2020;4(1):81-11. 33025309 10.1186/s41687-020-00249-y PMC7538479

[19] Aaronson NK , Ahmedzai S, Bergman B, et al The European Organization for Research and Treatment of Cancer QLQ-C30: a quality-of-life instrument for use in international clinical trials in oncology. J Natl Cancer Inst. 1993;85(5):365-376. 8433390 10.1093/jnci/85.5.365

[20] Cull A , Aaronson NK, Ahmedzai S, et al The European Organization for Research and Treatment of Cancer (EORTC) modular approach to quality of life assessment in oncology: an update. Qual Life Newsl. 1995;13:1-2.

[21] Fayers PA , Bjordal K, Groenvold M, Curran D, Bottomley A. EORTC QLQ-C30 Scoring Manual. 3rd ed. Brussels: European Organisation for Research and Treatment of Cancer; 2001.

[22] Husson O , de Rooij BH, Kieffer J, et al The EORTC QLQ‐C30 summary score as prognostic factor for survival of patients with cancer in the “real‐world”: results from the population‐based PROFILES registry. Oncologist. 2020;25(4):e722-e732. 32297435 10.1634/theoncologist.2019-0348 PMC7160310

[23] Cohen J. Statistical Power Analysis for the Behavioral Sciences. 2nd ed. Hillsdale, NJ: L. Erlbaum Associates; 1988.

[24] Rijksoverheid. Discussienota: Zorg voor de Toekomst. Den Haag: Ministerie van Volksgezondheid WeS; 2020.

[25] World Health Organization. Global health workforce statistics. https://www.who.int/health-topics/health-workforce#tab=tab_1. Accessed July 19, 2022.

[26] Nipp RD , Horick NK, Deal AM, et al Differential effects of an electronic symptom monitoring intervention based on the age of patients with advanced cancer. Ann Oncol. 2020;31(1):123-130. 31912785 10.1016/j.annonc.2019.09.003 PMC7497788

[27] Baratelli C , Turco CGC, Lacidogna G, et al The role of patient-reported outcomes in outpatients receiving active anti-cancer treatment: impact on patients’ quality of life. Support Care Cancer. 2019;27(12):4697-4704. 30949832 10.1007/s00520-019-04777-2

[28] Islam KM , Anggondowati T, Deviany PE, et al Patient preferences of chemotherapy treatment options and tolerance of chemotherapy side effects in advanced stage lung cancer. BMC Cancer. 2019;19(1):835. 31455252 10.1186/s12885-019-6054-x PMC6712837

[29] Lehto RH. Symptom burden in lung cancer: management updates. Lung Cancer Manag. 2016;5(2):61-78. 30643551 10.2217/lmt-2016-0001 PMC6310300

[30] Iyer S , Taylor-Stokes G, Roughley A. Symptom burden and quality of life in advanced non-small cell lung cancer patients in France and Germany. Lung Cancer. 2013;81(2):288-293. 23561304 10.1016/j.lungcan.2013.03.008

[31] Demedts I , Himpe U, Bossuyt J, et al Clinical implementation of value based healthcare: impact on outcomes for lung cancer patients. Lung Cancer. 2021;162:90-95. 34763159 10.1016/j.lungcan.2021.10.010

[32] CBS. Internettoegang en Internetactiviteiten; Persoonskenmerken. https://opendata.cbs.nl/#/CBS/nl/dataset/84888NED/table. Accessed October 21, 2022.

[33] Cocks K , King MT, Velikova G, et al Evidence-based guidelines for interpreting change scores for the European Organisation for the Research and Treatment of Cancer Quality of Life Questionnaire Core 30. Eur J Cancer. 2012;48(11):1713-1721. 22418017 10.1016/j.ejca.2012.02.059

