# Supplementary material for: Quality of life after patient-initiated vs physician-initiated response to symptom monitoring: the SYMPRO-Lung trial
Source: J Natl Cancer Inst. 2023 Aug 21;115(12):1515–25. doi: 10.1093/jnci/djad159 (PMC10699799; doi:10.1093/jnci/djad159)
Supplement: djad159_Supplementary_Data [file djad159_supplementary_data.pdf]

Supplementary Materials

Supplementary Methods

Primary outcomes

The Summary score was calculated as the mean of the combined scales of the QLQ-C30 scale scores (excluding financial impact and a two-item Global quality of life scale).[1] All scale scores were linearly transformed to 0-100, following EORTC guidelines. For both scales higher scores indicated improved functioning.

Cocks et al. guidelines were used to interpret the clinical relevance of within group change scores.[2] The Physical functioning thresholds for small and medium improvement were set at 2 to 7 and >7, respectively. The thresholds for small, medium and large deteriorations were set at -5 to -10, -10 to -17, and <-17 respectively. To date, no within group change score thresholds have been established for the Summary score; thus any level of change was investigated.

Secondary outcomes

The remaining QLQ-30 scales were chosen as our secondary outcomes. For the remaining functioning scales (Global health status, Role functioning, Emotional functioning, Cognitive functioning and Social functioning) higher scores indicated improved functioning. For the Symptom scales (fatigue, nausea and vomiting, pain, dyspnea, insomnia, appetite loss, constipation, diarrhea, and financial impact) higher scores indicated more symptoms/problems.[3] Likewise, Cocks guidelines were used to interpret clinically relevant changes.[2]

Sensitivity analyses

Sensitivity analyses were conducted to check the influence of missing values on our results. For this, we performed multiple imputation for those patients who had missing values at T1.[4] Missing patients were compared with non-missing patients, and between the control- and intervention group, to investigate if they were missing at random. Multiple imputation was based on patients with the same characteristics on age, gender, group, ECOG Performance Status (PS), histology, treatment, cancer stage and EORTC-QLQ-C30 scale scores on T0 and T1.

References

1. Pompili C, Koller M, Velikova G, et al. EORTC QLQ-C30 summary score reliably detects changes in QoL three months after anatomic lung resection for non-small cell lung cancer (NSCLC). *Lung Cancer*. 2018;123:149-154.

2. Cocks K, King MT, Velikova G, et al. Evidence-based guidelines for interpreting change scores for the European Organisation for the Research and Treatment of Cancer Quality of Life Questionnaire Core 30. *Eur J Cancer*. Jul 2012;48(11):1713-21. doi:10.1016/j.ejca.2012.02.059

3. Fayers P AN, Bjordal K, Groenvold M, Curran D, Bottomley A. EORTC QLQ-C30 Scoring Manual. 3rd ed. *Brussels: European Organisation for Research and Treatment of Cancer*. 2001;

4. Gunnes N, Seierstad TG, Aamdal S, et al. Assessing quality of life in a randomized clinical trial: correcting for missing data. *BMC Med Res Methodol*. Apr 30 2009;9:28. doi:10.1186/1471-2288-9-28

Supplementary Tables

Supplementary Table 1

Completion rates by study week

Active intervention group					Reactive Intervention group			
**Week**	**n**	**Completed**	**Rate**		**Week**	**n**	**Completed**	**Rate**
0	69	39	56.5%		0	35	33	94,3%
1	128	95	74,2%		1	61	55	90,2%
2	145	115	79,3%		2	82	66	80,5%
3	155	109	70,3%		3	87	61	70,1%
4	155	106	68,4%		4	86	62	72,1%
5	156	103	66,0%		5	88	60	68,2%
6	155	100	64,5%		6	88	58	65,9%
7	153	100	65,4%		7	88	54	61,4%
8	152	107	70,4%		8	86	60	69,8%
9	149	92	61,7%		9	86	52	60,5%
10	149	89	59,7%		10	86	56	65,1%
11	147	91	61,9%		11	86	52	60,5%
12	144	92	63,9%		12	85	57	67,1%
13	143	93	65,0%		13	85	55	64,7%
14	143	86	60,1%		14	85	54	63,5%
15	140	101	72,1%		15	83	59	71,1%
			66,2%					70,3%

Supplementary Table 2

Sensitivity analyses with multiple imputation for missing individuals for difference in mean change scores for multivariable linear regression health-related quality of life from baseline to 15 weeks intervention versus control group including confounders.

EORTC-QLQ-C30					Multivariable analyses			
**N = 515 (intervention 249, control 266)**		**Mean baseline**	**Mean 15 weeks**	**Mean change score and clinical relevance***	**B (SE)**	**95% CI**	***p*-value**	**ES**
Functioning Scales								
QLQ summary score	Intervention group	73.95	78.93	+4.98 n/a	3.87 (1.44)	1.03 – 6.71	0.008	0.42
	Control group	75.55	75.69	+0.14 n/a				
Physical functioning	Intervention group	71.22	72.62	+1.40 Trivial	6.03 (1.93)	2.26 – 9.81	0.002	0.56
	Control group	72.08	66.59	-5.49 Small deterioration				
Role functioning	Intervention group	61.78	63.81	+2.03 Trivial	7.36 (2.75)	1.95 – 12.76	0.008	0.36
	Control group	61.47	55.97	-5.50 Trivial				
Social functioning	Intervention group	71.35	76.78	+5.43 Small improvement	7.74 (2.64)	2.52 – 12.96	0.004	0.44
	Control group	73.62	70.60	-3.02 Trivial				
Symptom Scales								
Fatigue	Intervention group	40.38	35.78	+4.60 Small improvement	-7.18 (2.71)	-12.54 – -1.81	0.009	0.41
	Control group	41.14	42.22	-1.08 Trivial				
Dyspnea	Intervention group	33.87	34.31	-0.44 Trivial	-9.32 (2.91)	-15.04 – -3.60	0.001	0.53
	Control group	35.21	43.43	-8.22 Small deterioration				
Constipation	Intervention group	22.76	12.41	+10.35 Medium improvement	-7.41 (2.31)	-11.94 – -2.88	0.001	1.15
	Control group	15.79	15.76	+0.03 Trivial				

EORTC Quality of life score and functioning scales: higher score = better QOL/functioning. EORTC Symptom scales: higher score = worse symptoms.

Abbreviations: T1, 15 weeks; ES, Cohen's D effect size: 0.2 = small, 0.5 = medium, 0.8 = large. *P*-values for between group comparisons.
*Clinical relevance according to guidelines of Cocks et al. 2012 for longitudinal within-group differences.[2]
Multivariable analyses were controlled for histology, treatment, cancer stage, baseline score and transfer sequence of the hospitals.

Supplementary Figures

Supplementary Figure 1

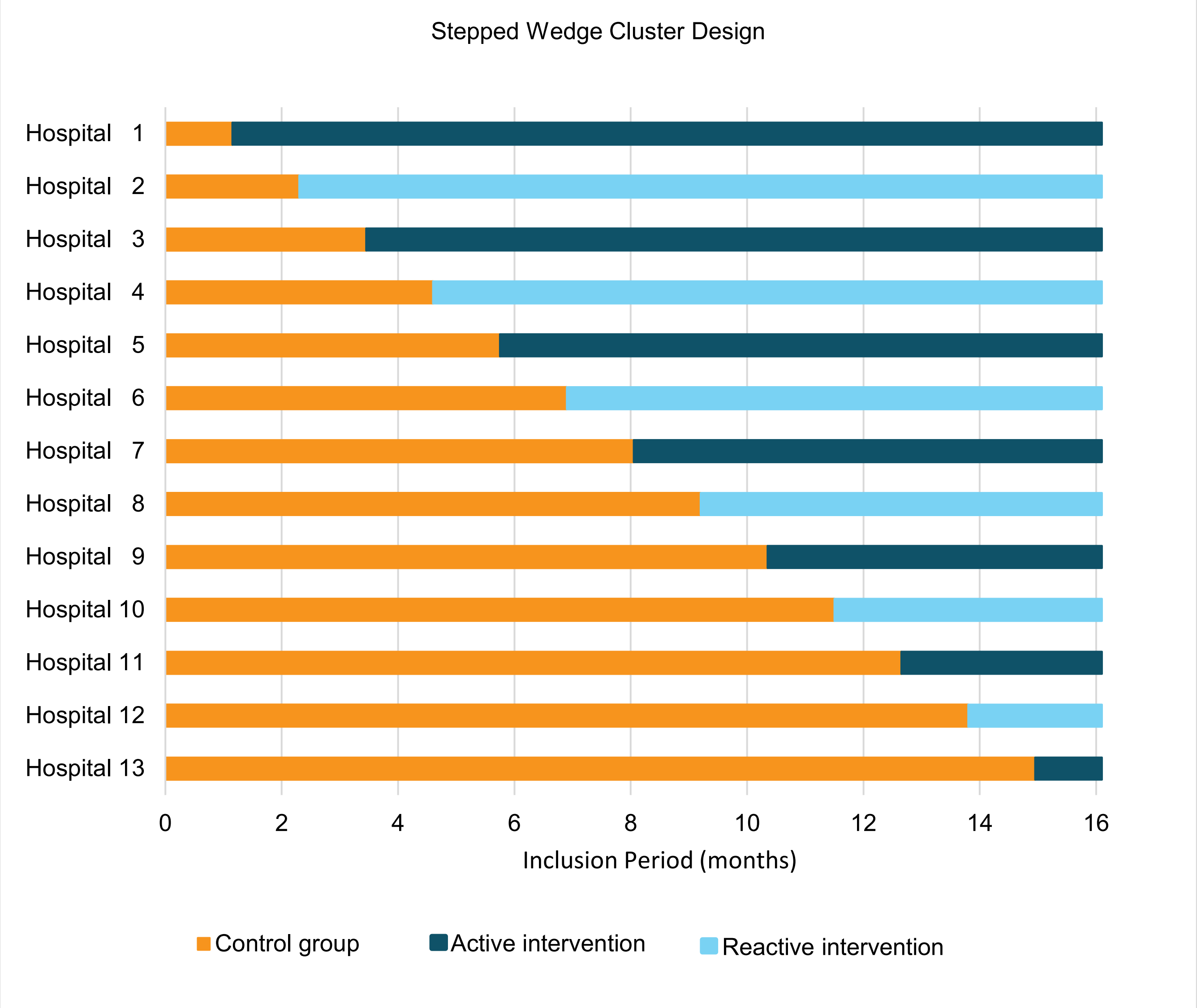

Supplementary Figure 2

Proportion of patients with health-related quality of life changes at 15 weeks (T1) compared with baseline

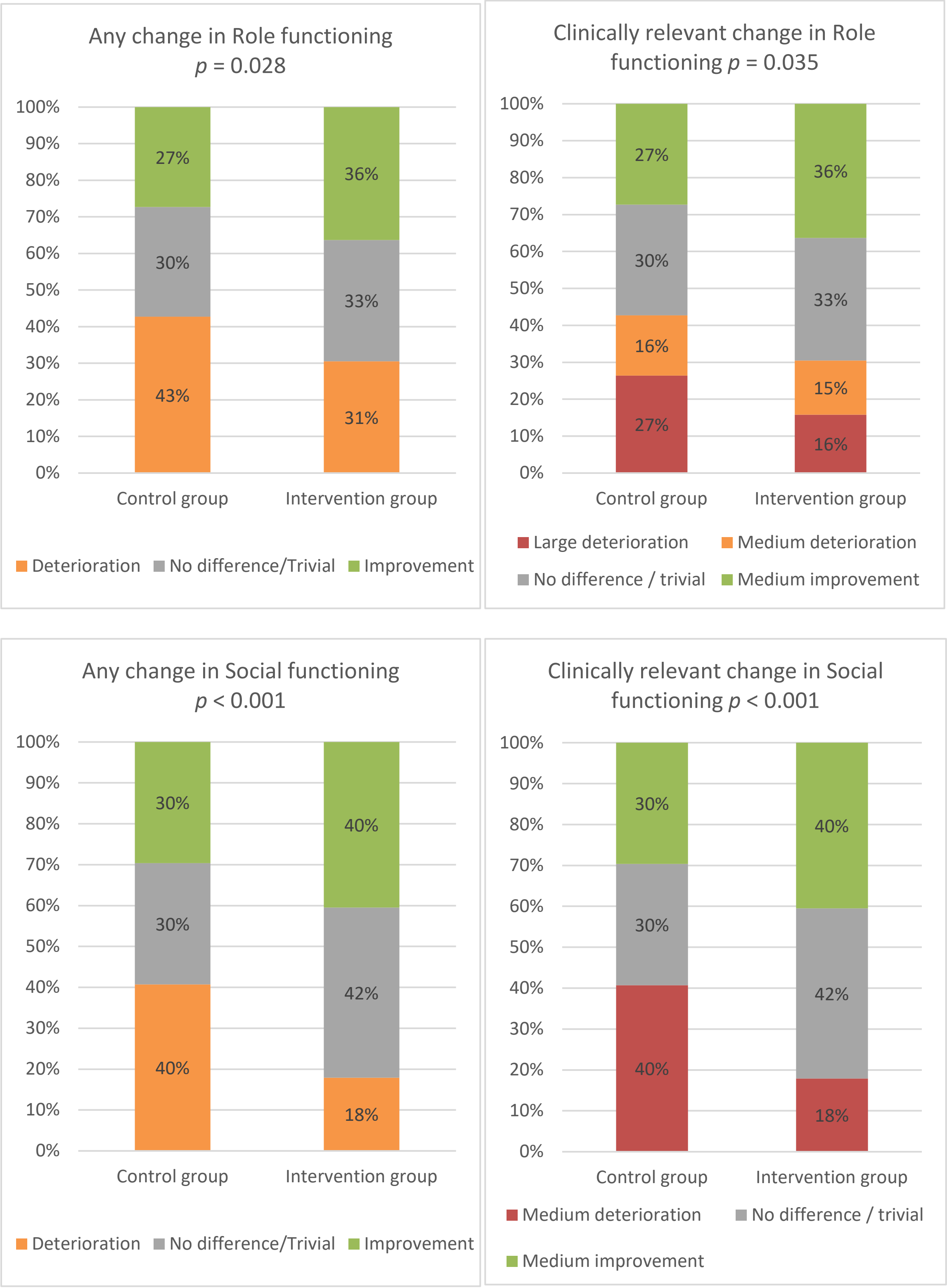

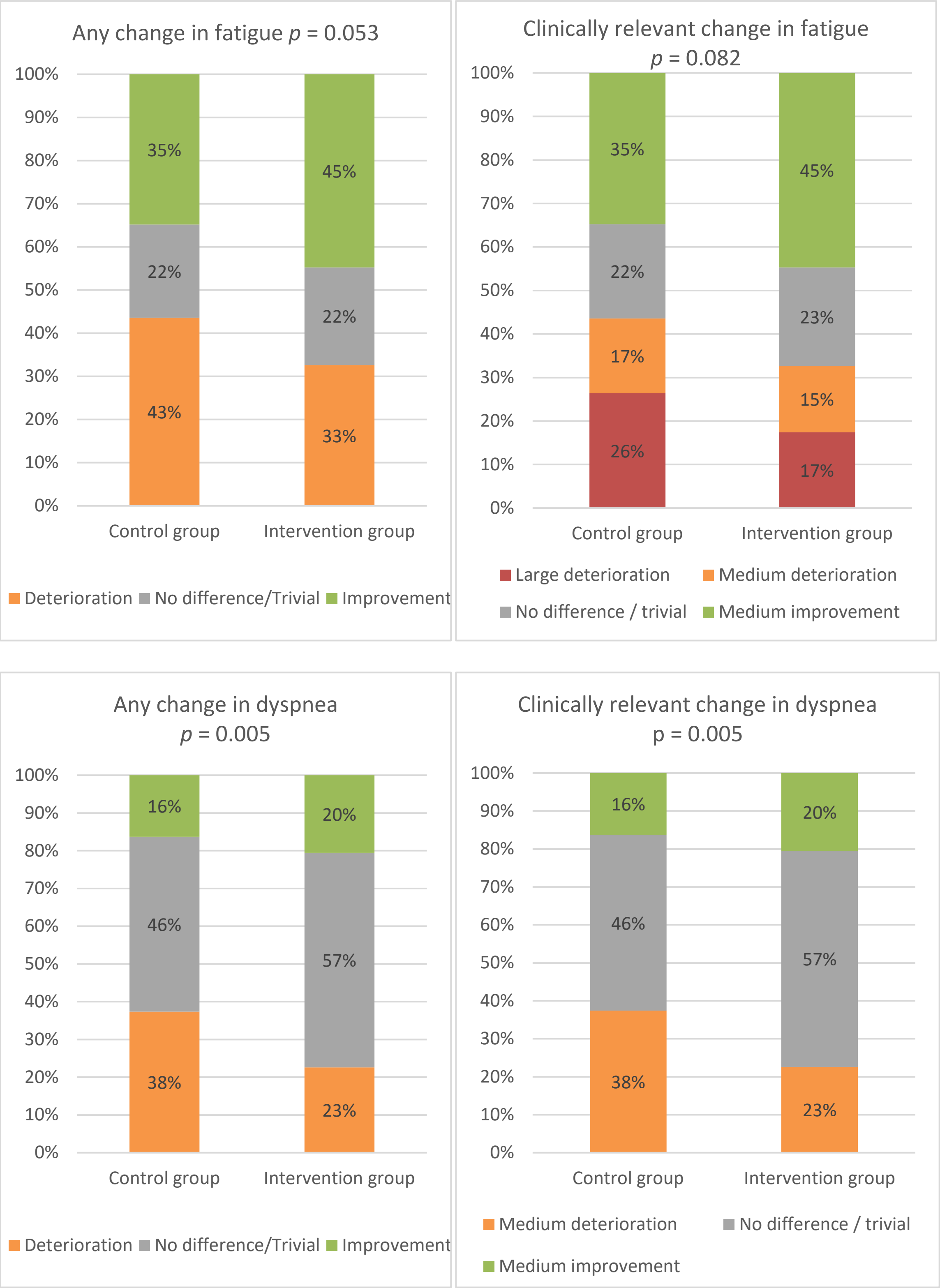
Any change in fatigue p = 0.053
100%
90%
80%
70%
60%
50%
40%
30%
20%
10%
0%
35%
22%
43%
45%
22%
33%
Control group
Intervention group
Deterioration
No difference/Trivial
Improvement
Clinically relevant change in fatigue
p = 0.082
35%
22%
17%
26%
45%
23%
15%
17%
Control group
Intervention group
Large deterioration
Medium deterioration
No difference / trivial
Medium improvement
Any change in dyspnea
p = 0.005
16%
46%
38%
20%
57%
23%
Control group
Intervention group
Deterioration
No difference/Trivial
Improvement
Clinically relevant change in dyspnea
p = 0.005
16%
46%
38%
20%
57%
23%
Control group
Intervention group
Medium deterioration
No difference / trivial
Medium improvement

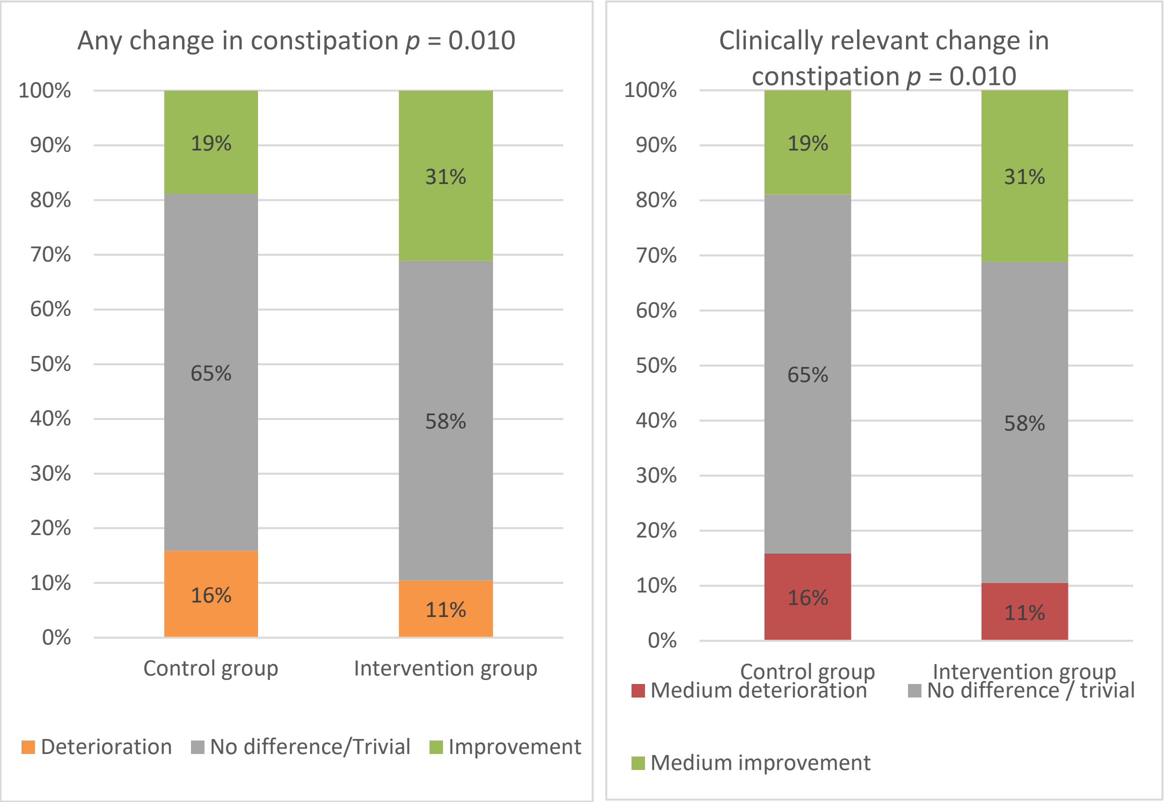

Proportion of patients with health-related quality of life changes at 15 weeks (T1) compared with baseline. For the clinically relevant changes Cocks' guidelines for within group changes were used:[2] Role functioning: Improvements >12 medium, 12-6 small; No difference/trivia 6 to -7; Deteriorations -7 to -14 small, -14 to -22 medium, <-22 large. Social functioning: Improvements >8 medium, 8-3 small; No difference/trivia 3 to -6; Deteriorations -11 to -6 small, <-11 medium. Fatigue: Improvements >-9 medium, -9 to -4 small; No difference/trivia -4 to 5; Deteriorations 5-10 small, 10-15 medium, <15 large. Dyspnea: Improvements >-9 medium, -9 to -2 small; No difference/trivia -2 to 5; Deteriorations 5-11 small, <11 medium. Constipation: Improvements >-10 medium, -10 to -4 small; No difference/trivia -4 to 5; Deteriorations 5-15 small, <15 medium. *P*-values were calculated using Pearson's Chi-Square tests comparing the control- versus the intervention group.
